# An Analysis of Regulatory T-Cell and Th-17 Cell Dynamics during Cytomegalovirus Replication in Solid Organ Transplant Recipients

**DOI:** 10.1371/journal.pone.0043937

**Published:** 2012-10-11

**Authors:** Adrian Egli, Moacyr Silva, Daire O'Shea, Leticia E. Wilson, Aliyah Baluch, Luiz F. Lisboa, Luis G. Hidalgo, Deepali Kumar, Atul Humar

**Affiliations:** 1 Alberta Transplant Institute and Li Ka Shing Institute of Virology, University of Alberta, Edmonton, Alberta, Canada; 2 Histocompatibility Laboratory, Department of Laboratory Medicine and Pathology, University of Alberta, Edmonton, Alberta, Canada; Rush University, United States of America

## Abstract

**Background:**

CMV-specific T-cells are crucial to control CMV-replication post-transplant. Regulatory T-cells (T-regs) are associated with a tolerant immune state and may contribute to CMV-replication. However, T-cell subsets such as T-regs and IL-17 producing T-cells (Th-17) are not well studied in this context. We explored T-regs and Th-17 frequencies during CMV-replication after transplantation.

**Methods:**

We prospectively evaluated 30 transplant patients with CMV-viremia. We quantified CMV-specific CD4^+^ and CD8^+^ T-cells, T-regs (CD4^+^CD25^+^FoxP3^+^) and Th-17 frequencies using flow-cytometry and followed patients requiring anti-viral treatment. Two subsets were compared: anti-viral treatment requirement (n = 20) vs. spontaneous clearance of viremia (n = 10).

**Results:**

Higher initial CMV-specific CD4^+^ T-cells and lower T-regs were observed in patients with spontaneous clearance (p = 0.043; p = 0.021 respectively). Using a ratio of CMV-specific CD4^+^ T-cells to T-regs allowed prediction of viral clearance with 80% sensitivity and 90% specificity (p = 0.001). One month after stop of treatment, the same correlation was observed in patients protected from CMV-relapse. The ratio of CMV-specific CD4^+^ T-cells to T-regs allowed prediction of relapse with 85% sensitivity and 86% specificity (p = 0.004). Th-17 responses were not correlated with virologic outcomes.

**Conclusions:**

This study provides novel insights into T-regs and Th-17 subpopulations during CMV-replication after transplantation. These preliminary data suggest that measurement of CMV-specific CD4^+^ T-cells together with T-regs has value in predicting spontaneous clearance of viremia and relapse.

## Introduction

After solid organ transplantation, cytomegalovirus (CMV)-replication may result in viral syndrome or tissue invasive disease [1]. CMV-replication may also play a role in acute and chronic allograft injury/rejection, impaired long-term graft outcomes, and increased rates of bacterial and fungal infection [1], [2]. The mechanisms by which these effects occur are incompletely characterized.

In the post-transplant setting the adaptive immune response, and specifically CD4^+^ and CD8^+^ T-cell responses play a prominent role in the control of CMV replication. A decrease in CMV-specific CD4^+^ and CD8^+^ T-cells has been associated with progressive CMV-replication [3], [4], [5], [6], [7], [8]. More recently, additional T-cell subsets have been recognized to have important roles [9]. For example, internal regulatory mechanisms such as regulatory T-cells (T-regs; CD4^+^CD25^+^FoxP3^+^) may modify CMV-specific CD4^+^ and CD8^+^ T-cell functions leading to an increased risk for progressive CMV-replication [10]. T-regs primarily function through the release of inhibitory cytokines such as IL-10 and TGF-β [10]. T-regs play an important role in maintaining self-tolerance and are being studied as a potential means to promote an immunotolerant state post-transplant [11]. Following liver transplantation, high frequencies of T-regs in peripheral blood and hepatic tissue were associated with a more aggressive recurrence of hepatitis C virus [12].

IL-17 producing CD4^+^ T-cells (Th-17) are a newly described subtype of CD4^+^ T-cells [13]. They constitute a part of the normal host response to infection. Due to their pro-inflammatory effect, Th-17 cells have also been associated with allograft rejection and autoimmune disease [14]. The precise role of Th-17 responses during CMV-replication has not been well elucidated, although recent studies suggest that various viral infections such as murine CMV, influenza virus and herpes simplex virus induce a Th-17 response [15], [16], [17].

Following transplantation, patients are typically monitored with molecular diagnostic tools to detect CMV-replication at an early stage. The kinetics of virus replication however, is only weakly associated with future outcomes such as progressive replication and the development of tissue-invasive disease. Novel immunological biomarkers, such as virus-specific T-cell responses might bridge this gap in our knowledge. The full repertoire of T-cell responses including absolute T-regs and Th-17 subsets has not been studied in transplant recipients with active CMV-replication. We hypothesized that following transplantation significant alterations occur in T-regs and Th-17 dynamics in the setting of CMV-replication. The present study aimed to prospectively assess CMV-specific CD4^+^ and CD8^+^ T-cells, total T-regs, and Th-17 frequencies in transplant recipients with concurrent CMV-replication. These immunological parameters were assessed simultaneously with clinical parameters such as CMV viral-load, kinetics of clearance, and relapse rates. We aimed to use those markers to predict clearance at onset of viremia and relapse after stop of treatment.

## Materials and Methods

### Ethics Statement

The study was approved through the University of Alberta Ethics Review Board and all patients provided written informed consent.

### Patient population

Adult solid organ transplant patients with CMV-replication (defined as viremia >500 copies/mL of plasma) were prospectively identified and enrolled. Blood samples for immunological assay were collected within three days of detection of viral replication. Patients, who commenced antiviral treatment, underwent follow-up blood sampling at regular time intervals (see below, **Figure S1**). Those who did not start treatment only had an initial sample drawn at the time of first viremia detection, unless later in the follow-up anti-viral treatment was required. Clinical outcomes and follow-up data were collected for a minimum of six months post-enrollment. The decision to use anti-viral treatment and the type of therapy used was at the discretion of the treating physician. The management of patients was guided by the local institutional protocol in which patients with low-level viremia (<10,000 copies/mL) are managed by surveillance without antiviral treatment. Patients with symptoms or with higher viral-loads received antiviral therapy with either intravenous ganciclovir or oral valganciclovir until resolution of viremia.

### Definitions

Definitions of CMV-disease were based on recommendations for use in clinical studies including viral syndrome and tissue invasive disease [18]. Virologic relapse was defined as clearance of initial viremia followed by subsequent detection of virus on at least two separate occasions; or in patients with a minimum 2-log_10_ increase in viral-load after treatment. Clinical signs and symptoms of relapse were assessed for a minimum of six months after the initial episode of disease.

### Viral-load measurements

CMV viral-load testing was performed on plasma samples using an in-house real-time light-cycler based PCR assay that has been validated for clinical use [19]. In this assay, DNA was extracted from 200 µl of plasma and amplified using primers targeting a conserved region of the glycoprotein B gene of CMV, and detection achieved using fluorescent probes as previously described [see ref 19 for primer and probe sequences]. The assay has a lower limit of quantification of 500 copies/mL and a dynamic range of approximately 6-log_10_ copies [20] and has been validated against the commercially available COBAS Amplicor CMV Monitor assay. After initial detection, all patients had weekly viral-load testing performed. For patients who commenced anti-viral treatment, individual viral kinetic calculations were performed. Viral-load was plotted against time and best-fit exponential decay curves were applied to generate appropriate mathematical descriptions of virologic outcomes [21]. The slope of the decay curve was calculated based on this best-fit line. In this context, the more negative the decay slope, the more rapid the viral clearance. Positive initial decay slopes indicate increasing viral-loads co-incident with the initiation of treatment.

### Measurement of virus-specific CD4^+^ and CD8^+^ T-cells, T-regs, and Th-17-cells

Cellular immune responses in patients commenced on antivirals were measured at three time-points: the onset of viremia (synonymous with the initiation of anti-viral therapy); end of treatment; and one month after treatment completion. In patients who did not receive anti-viral therapy, T-cell responses were only measured at the onset of viremia. We quantified CMV-specific CD4^+^ and CD8^+^ T-cells according to previously published protocols with slight modifications [4]. Briefly, peripheral blood mononuclear cells (PBMCs) were isolated from whole blood using Ficoll-Paque (Pharmacia, Uppsala, Sweden) gradient density centrifugation and stored in liquid nitrogen until use. For determination of CMV-specific CD8^+^ T-cells responses, 1×10^6^ PBMCs were stimulated for 16 hours with peptide pool libraries covering the entire IE-1 (immediate early-1), pp72 and pp65 proteins. Each peptide is 15mer-long with an 11 amino acid overlap; final concentration 2 µg/mL in DMSO (JPT Technologies, Berlin, Germany). DMSO concentration in all experiments was maintained less than 1%. For CMV-specific CD4^+^ T-cell responses, 1×10^6^ PBMCs were stimulated for 16 hours with CMV whole virus lysate derived from fibroblast cultures (Advanced Biotechnologies Inc., Columbia, MD) at a concentration of 3 µg/mL. Selection of type of stimulant [peptide vs. viral lysate], concentration used, and duration of stimulation was based on validation studies performed using CMV seropositive and seronegative healthy volunteers to optimize the assays. We found that that viral lysate was optimum for stimulation of CD4+ T cells whereas a peptide pool optimally stimulated CD8+ T-cells. This is also supported by the literature [4], [22]. Anti-CD3 was used as a positive control. Media alone served as a negative control. Two hours following stimulation, Brefeldin A at a final concentration of 10 µg/mL was added to block interferon-gamma (IFN-γ) secretion. Next the PBMCs underwent cell surface staining. Following fixation and membrane perforation using standard buffers (eBiosciences), intracellular staining was performed. The following fluorescent monoclonal antibodies were used: phycoerythrin-Cy7 (PE-Cy7) anti-human CD4 (L3T4), allophycocyanin-eFluor™ 780 anti-human CD8 (CD8a; alpha chain), PE-anti-human interferon-gamma (IFN-γ) (all eBioscience Inc., San Diego, CA). Isotype antibodies were used as appropriate.

T-regs were defined as CD4^+^CD25^+^FoxP3^+^ T-cells using anti-human CD25 (APC) and anti-human Foxp3 (PE). Th-17 cells were identified as CD4^+^IL17^+^ T-cells using anti-human IL-17A (PE, Interleukin-17A). IL-17 production was induced by 16 h stimulation with PMA (phorbol myristate acetate) and ionomycin at final concentrations of 1 µg/mL and 500 ng/mL respectively at 37°C and 5% CO_2_ as previously published [23].

Flow data were acquired using a BD FACSArray™ Bioanalyzer System equipped with FACSArray™ System Software and results were analyzed using FCS Express Version 3 (De Novo Software, Los Angeles, CA, USA). 100,000 CD4^+^ T-cells were obtained per sample.

#### Statistical analysis

The CMV-specific CD4^+^ T-cell response is expressed as the amount of IFN-γ producing CD4^+^ T-cells (as a percentage of total CD4^+^ T-cells) having subtracted the respective negative controls. CMV-specific CD8^+^ T-cell responses are expressed in the same manner. T-regs (CD4^+^CD25^+^FoxP3^+^ T-cells) and Th-17 cells (IL-17 producing CD4^+^ T-cells) are both expressed as a percentage of total CD4^+^ T-cells. Responses <0.2% CMV-specific CD4^+^ and CD8^+^ T-cells were considered to not be significantly different from the negative control (based on calculating two standard deviations from the mean negative control value). Statistical analyses were performed using SPSS (version 18.0, Chicago, Ill.) and GraphPad Prism (version 4.0, La Jolla, CA). Fisher's exact test or Chi-square test was used for categorical variables and the Mann-Whitney U test was used for continuous variables. Correlations were performed using the Spearman correlation coefficient. Alpha was set equal to 0.05, and all tests of significance were two tailed.

## Results

### Patient population

The clinical and demographic characteristics of the enrolled patients are listed in Table 1. A total of 30 patients were enrolled including 20 patients with high-level viremia and/or symptomatic CMV-disease who required the commencement of anti-viral therapy. This group was compared to 10 patients with asymptomatic low-level viremia, who spontaneously cleared CMV viremia without treatment. The median time to CMV-replication post-transplant was 151 days (range 26–644 days). The majority (93.3%) of patients were receiving immunosuppression with a calcineurin inhibitor [tacrolimus or cyclosporine], prednisone and mycophenolate mofetil (MMF) at the time of initial CMV detection. No patient was on sirolimus [Table 1]. Previous anti-CMV prophylaxis had been administered to 73.3% of patients. No patient had biopsy proven acute rejection within a three-month period of follow-up after the initial detection of CMV. No statistically significant difference in CMV serological status was present between patient groups. There was a higher percentage of D+/R− patients in the progressive viremia group vs. the spontaneous clearance group (45% vs. 30%; p = 0.69) although this was not statistically significant. No significant difference in type of immunosuppression or transplanted organ was observed between the two groups [p = n.s.].

**Table 1:** Baseline characteristics of consecutively recruited 30 patients with CMV-viremia.

Variable	All, n = 30	Progressive viremia, n = 20	Spontaneous clearance of viremia, n = 10
Gender, male/female	23/7	15/5	8/2
Age in years, median (range)	49 (26–75)	60 (26–70)	57.5 (42–75)
Type of transplant			
Kidney	12 (40.0%)	6	6
Lung	11 (36.7%)	7	4
Heart	3 (10%)	3	0
Liver	2 (6.7%)	2	0
Kidney-Pancreas	2 (6.6%)	2	0
Immunosuppression			
Tac/Pred/MMF	22 (73.3%)	15	7
CsA/Pred/MMF	6 (20%)	4	2
Others[Tac/MMF, Tac/Pred]	2 (6.7%)	1	1
Tacrolimus trough levels, median (range); initial time-point	7.75 (4.4–16.4)	8.4 (4.4–16.4)	7.7 (5–12.2)
Induction			
ATG	5	2	3
others	7	6	1
CMV serostatus			
D+/R−	12 (40)	9	3
R^+^	18 (60)	11	7

Immunosuppression: Tac, tacrolimus; MMF, mycophenolate mofetil; CsA, cyclosporine; Pred, prednisolone; ATG, anti-thymocyte globulin. CMV serostatus: D, donor; R, recipient; +, positive; − negative. All comparisons were not significant.

### CMV viral-loads and kinetics

Ten patients spontaneously cleared CMV-viremia. Median viral-load at baseline in these patients was 2.77 log_10_ copies/mL (range <2.6–3.92 log_10_ copies/mL); and median time to viral clearance was 7 days (range 7–18 days). Twenty patients received antiviral therapy and the following viral-clearance kinetics was observed: median viral-load at baseline was 4.6 log_10_ copies/mL (range 3.86–6.17 log_10_ copies/mL) and the median time to clearance was 28 days (range 19–74 days). One patient did not achieve complete clearance of viremia despite prolonged antiviral therapy. Seven patients (35.0%) had relapse of detectable CMV-viremia during the follow-up period.

### Cytomegalovirus-specific CD4^+^ and CD8^+^ T-cell responses

**Figure 1:**
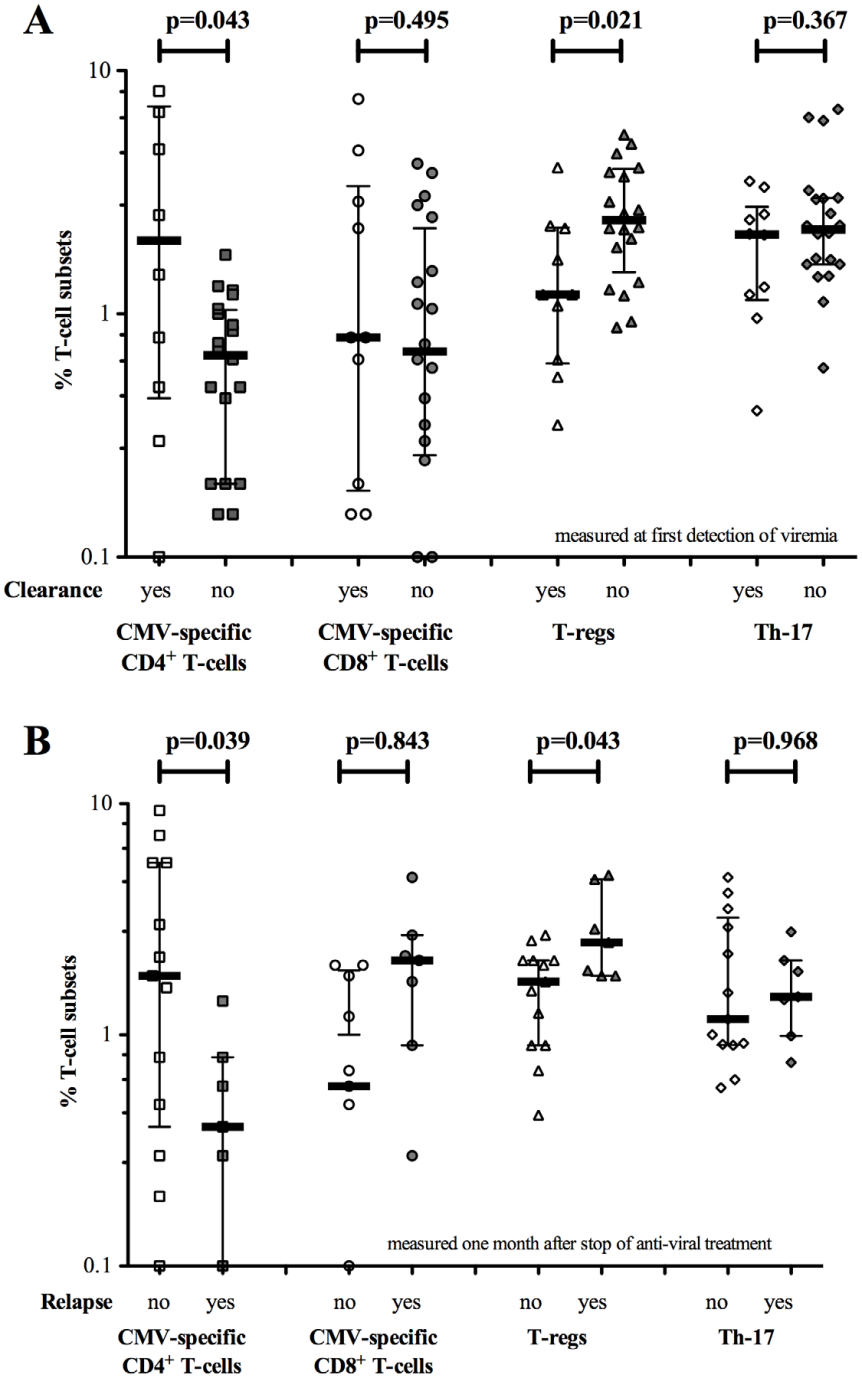
CMV-specific CD4^+^ and CD8^+^ T-cell, T-reg and Th-17 dynamics in patients with different viral outcomes. Square (□) indicates CMV-specific CD4^+^ T-cell response. Circle (○) indicates CMV-specific CD8^+^ T-cell response. Triangle (▵) indicates T-reg response. Diamond (⋄) indicates Th-17 response. Black bar indicates median value, whiskers indicate inter-quartile range. (**A**), patients with spontaneous clearance of viremia compared with patients with progressive viral-loads who commenced treatment. Initial immune response at treatment commencement was determined. (**B**), patients with relapse compared to patients without relapse. Immune responses as determined 1 month after treatment discontinuation.

The baseline CMV-specific CD4^+^ and CD8^+^ T-cell responses were determined in 30 patients at the onset of viremia. For 20 patients this was also the time-point of treatment initiation. The median fraction of CMV-specific CD4^+^ and CD8^+^ T-cells of the total cohort was 0.88% (range 0.00–11.00%, n = 30), and 0.78% (range 0.00–7.65%, n = 30) respectively. 23 patients (76.7%) showed an initial positive CMV-specific CD4^+^ T-cell response (>0.2%), whereas 18 patients (60%) showed an initial positive CMV-specific CD8^+^ T-cell response (>0.2%). Patients with CMV disease/progressive viremia had a significantly lower initial CMV-specific CD4^+^ T-cell frequency compared to patients with spontaneous clearance of viremia (0.68% vs. 2.00%; p = 0.043) (**Figure 1**).

**Figure 2:**
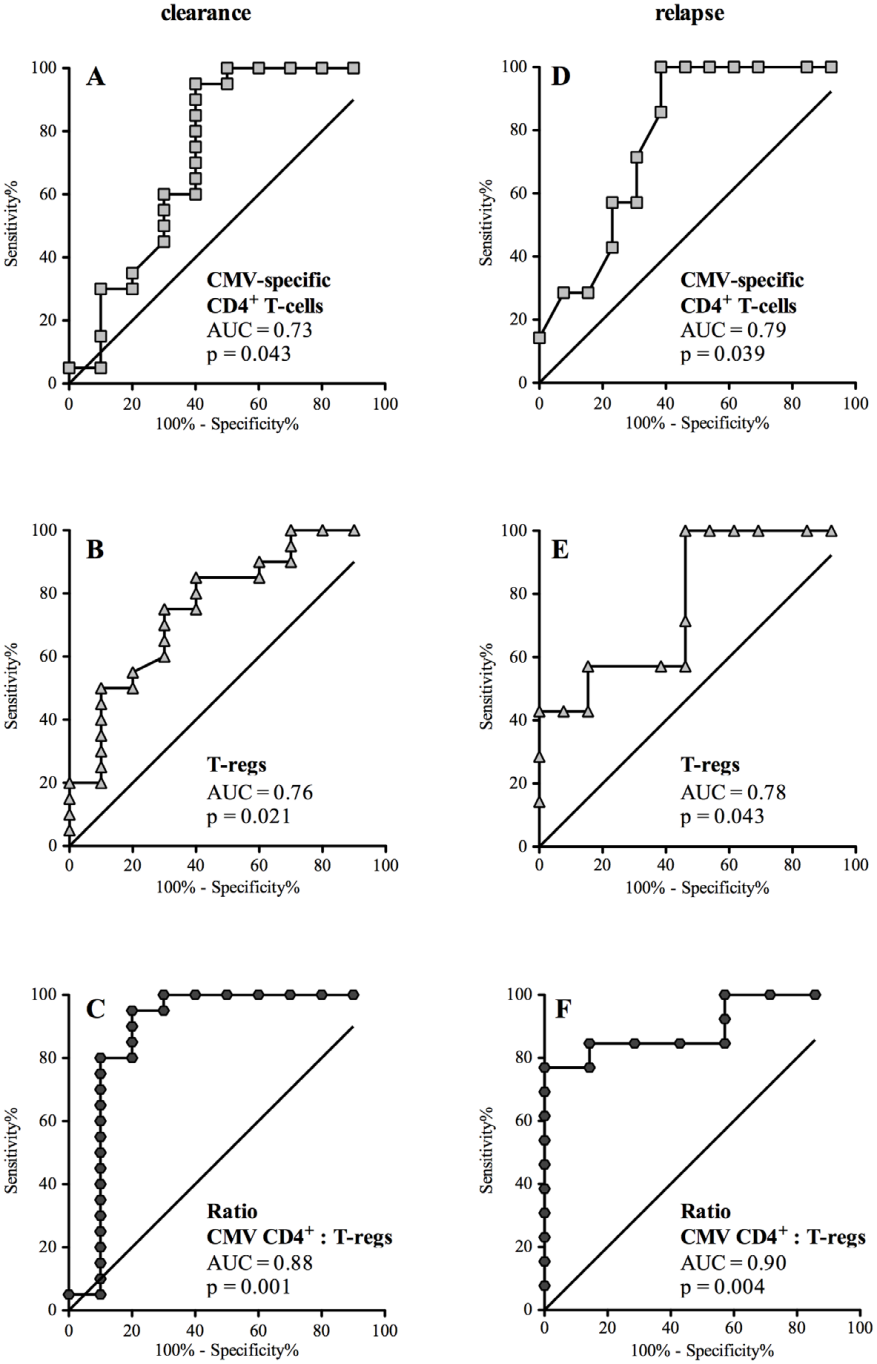
Receiver operating characteristic (ROC) curve analysis of CMV-specific CD4^+^ T-cells and T-regs to predict virus clearance and development of relapse. ROC curves were generated by analyzing the sensitivity and specificity of different cut-points for defining a positive test result and their ability to predict the outcome of interest. For Figure 2A, 2B, and 2C, the outcome of interest is spontaneous clearance of viremia [vs. progression]; n = 30. For Figure 2D, 2E, and 2F, the outcome is relapse of CMV after completion of treatment [n = 20]. The following three tests were analyzed: CMV-specific CD4^+^ T-cell response [Figure 2A and 2D], T-reg response [Figure 2B and 2E] and the ratio of the CMV-specific CD4^+^ T-cells to T-reg response [Figure 2C and 2F]. To determine the sensitivity and specificity for predicting clearance of viremia we used baseline immune measurements (2**A, 2B, and 2C**); for prediction of relapse we used measurements one month after completing treatment (**2D, 2E, and 2F**).

Receiver operating characteristic (ROC) curves were made to determine the potential clinical utility of these assays to predict clinical outcomes [Figure 2]. The outcomes analyzed were 1) spontaneous clearance of viremia [vs. progression] and 2) relapse of viremia after completion of treatment. For each outcome, different cut-offs for defining a positive T-cell measurement were analyzed for their sensitivity and specificity. For CD4^+^ T-cells, ROC analysis revealed that a cut-off of >1.4% CMV-specific CD4^+^ T-cells could predict spontaneous clearance of viremia with a 95% sensitivity and 60% specificity (AUC 0.73, p = 0.04) (**Figure 2A**).

The initial levels of CMV-specific CD4^+^ T-cells also predicted subsequent viral-loads and dynamics. The initial CMV-specific CD4^+^ T-cell frequency correlated significantly with CMV viral kinetics between days 7 and 14; expressed as the slope of viral decline (R^2^ = 0.27; p = 0.044). A trend was observed between the initial CMV-specific CD4^+^ T-cell count and subsequent viral-loads (i.e. two weeks later) (logistic regression R^2^ = 0.12; p = 0.06).

Patients who required antiviral therapy were further immunologically monitored at the point of treatment discontinuation and one month later to determine immunological risk markers to predict relapse after stopping treatment. Median CMV-specific CD4^+^ T-cell frequency at treatment discontinuation and 1 month later were 0.95% (range 0.00–6.20) and 0.80% (range 0.00–9.35), respectively. Relapse of CMV-viremia occurred in 7/20 (35%) patients at a median of 55 days following completion of antiviral therapy. Virologic relapse was symptomatic in 4 and asymptomatic in 3 patients. Patients with CMV-relapse displayed significantly lower CMV-specific CD4^+^ T-cell counts one month after treatment discontinuation compared with patients without relapse (0.40% vs. 1.80%; p = 0.039; **Figure 1** and **Table 2**). ROC analysis revealed that a cut-off of <1.5% CMV-specific CD4^+^ T-cells could predict relapse with 100% sensitivity and 61.5% specificity (AUC 0.79, p = 0.039) (**Figure 2D**). Patients without relapse exhibited a consistent increase in CMV-specific CD4^+^ T-cells over the follow-up period (0.50%; 1.15%; and 1.80% respectively); whereas in patients with CMV-relapse the opposite was observed (1.00%; 0.70% and 0.40% respectively) (**Figure S2**).

**Table 2:** Assessment of virologic relapse and its relation to T-cell subsets.

Variable		No relapse	Relapse	p-value
		Median (range), n = 13	Median (range), n = 7	
CMV-specific CD4^+^ T-cells (%)	End of treatment	1.15 (0.00–6.20)	0.70 (0.20–1.8)	0.937
	+1 month	1.80 (0.10–9.35)	0.40 (0.00–1.40)	0.039
CMV-specific CD8^+^ T-cells (%)	End of treatment	0.70 (0.05–8.65)	1.45 (0.1–3.4)	0.428
	+1 month	1.80 (0.05–10.60)	1.70 (0.3–4.75)	0.843
T-regulatory (%)	End of treatment	2.24 (0.58–6.37)	4.01 (0.88–4.91)	0.501
	+1 month	1.70 (0.45–2.7)	2.51 (1.8–4.9)	0.043
Th-17 (%)	End of treatment	2.10 (0.59–5.31)	2.18 (1.47–4.89)	0.552
	+1 month	1.17 (0.59–4.80)	1.46 (0.76–2.79)	0.968

Patients with progressive viremia (n = 20) at treatment discontinuation and 1 month later were analyzed. All statistical comparisons performed using Mann-Whitney Test.

### T-regulatory responses

Baseline T-regs percentages in 30 patients were median 2.24% [range 0.35–10.6%]. Baseline T-reg frequencies were significantly higher in patients with progressive viremia compared to those with spontaneous clearance of viremia (median 2.43% vs. 1.20% respectively; p = 0.02) (**Figure 1**). ROC analysis revealed a cut-off of <2.5% T-regs which predicted spontaneous clearance of viremia with 50% sensitivity and 90% specificity (AUC 0.76, p = 0.02) (**Figure 2B**). Interestingly, initial T-reg counts were significantly associated with viral-loads measured two weeks later (logistic regression R^2^ = 0.15; p = 0.038). In addition, a very strong association was observed between the initial T-regs frequencies and the slope of viral kinetic response between days 7 and 14 (R^2^ = 0.61; p<0.005). Median T-regs at treatment discontinuation and 1 month later were 2.51% (range 0.58–6.37) and 1.95% (range 0.45–4.90), respectively. Patients with relapsing CMV had significantly higher T-regs counts one month after stopping treatment compared with patients without relapse (2.51% vs 1.70%; p = 0.04; **Figure 1** and **Table 2**). No significant correlation was observed between concurrently measured CMV-specific CD4^+^ and CD8^+^ T-cell, T-regs, or Th-17 frequencies (p = n.s.; Spearman's rho).

### Combination of CMV-specific CD4^+^ T-cells and T-regs

Using a combination of CMV-specific CD4^+^ T-cells and T-regs significantly increased sensitivity and specificity to predict clearance and relapse. A ratio of CMV-specific CD4^+^ T-cells to T-regs (CD4^+^ T-cell: T-reg) above 0.4 could predict spontaneous clearance of viremia with 80% sensitivity and 90% specificity (AUC 0.88, p = 0.001) (**Figure 2C**). In addition, one month after antiviral discontinuation, the ratio of CMV-specific CD4^+^ T-cells to T-regs (CD4^+^ T-cell: T-reg) was highly predictive of relapse (AUC 0.90; p = 0.004) (**Figure 2F**).

### Th-17 responses

The baseline median Th-17 population in 30 patients was 2.14% (range 0.40% to 6.94%). No difference in Th-17 frequencies was observed between patients with spontaneous clearance of viremia and those with progressive viremia (p = n.s.; **Figure 1**). Additionally, no difference was observed in relation to relapsing CMV. The frequency of Th-17 cells was slightly reduced one month after treatment discontinuation (median 1.44%; p = n.s.).

## Discussion

Our study analyzed CMV-specific CD4^+^ and CD8^+^ T-cells in conjunction with T-regs and Th-17 frequencies. These were prospectively assessed in the context of viral kinetic monitoring in two different subgroups of patients with CMV-replication: a group with high viral-loads who commenced anti-viral treatment vs. a group who spontaneous clearance of viremia. We found that CMV-specific CD4^+^ T-cells and total T-regs are significantly associated with spontaneous clearance of viremia as well as with protection from relapse; and both immune markers used in combination enabled the prediction of virologic outcomes with >80% sensitivity and specificity. In addition, Th-17 cells were stably expressed and did not correlate with virologic outcomes. ROC curve analysis has been used to determine cutoffs for prediction of disease that have optimal sensitivity and specificity. We found that the best predictor of both spontaneous clearance of viremia and relapse after treatment was the frequency ratio of CMV CD4+ T cells: T-reg. The area under the curve approached 1 and was highly significant. Future validation can be done using these cutoff values.

Several studies have analyzed the predictive value of CMV-specific CD4^+^ and CD8^+^ T-cells with regards to the development of progressive CMV-replication and -disease [3], [4], [6], [7], [8], [22], [24], [25], [26]. Most, but not all, have concluded that a reduction in frequency of these cells is associated with an increased risk for CMV-replication. In our study, we did not observe a correlation between CMV-specific CD8^+^ T-cell responses and virologic outcomes. This may be due to the high proportion of R+ patients (60%) in our cohort. CMV-specific CD8^+^ T-cell responses are shown to be more prominent in D^+^R^−^ patients with primary infection [3], [4], [6]. However, in R+ patients, data on the role of CD8^+^ T cells in CMV control are not consistent [4], [7], [8], [22].

Previous studies have illustrated the importance of CMV-specific CD4^+^ T-cells in the long-term control of CMV in R^+^ patients [4], [7], [8]. Egli et al. reported that high pp65-specific CD4^+^ T-cell responses in kidney transplant patients were associated with a lower risk of both concurrent and future CMV-replication during an eight-week period following analysis [4]. Sester et al. determined that CMV-lysate specific CD4^+^ T-cells above 0.25% were protective against CMV-replication. In our study, we found that CMV-specific CD4^+^ T-cells greater than 1.4% were associated with spontaneous resolution of viremia, whilst a value below 1.5% (one month after treatment discontinuation) was associated with an increased risk of relapse. A positive correlation was also found between the frequency of CMV-specific CD4^+^ T-cells and the speed of viral decay. Stable CMV-specific CD4^+^ T-cell responses have been associated with control of late CMV reactivation in transplant recpient [4], [8], [22]; whereas CMV-specific CD8^+^ T-cells seem to be more important in primary infection and during early phases after transplantation [3], [6].

Increased frequencies of T-regs have been observed in a variety of infectious conditions. This can occur in acute infections, but is predominantly observed in chronic viral infections. During chronic infection, T-regs may play two potential roles: a beneficial role limiting collateral tissue damage, and a detrimental role impairing antiviral immune responses [27], [28]. Virus-specific T-regs have rarely been examined, but it is likely that the whole T-reg population contains at least some virus-specific cells. These populations are probably generated in parallel with virus-specific effector T-cell responses. This concept has recently been illustrated in a coronavirus infection model [29] and may represent a mechanism that serves to protect against an excessive immune response post exposure. Other acute viral infection models provide further support to this proposed role of T-regs in limiting immune-mediated pathology e.g. West Nile virus, respiratory syncytial virus, and influenza A virus replication [30], [31], [32].

An increase in T-reg numbers would be expected to lead to impaired clearance of the infecting pathogen. Evidence for this hypothesis has been found in several models, mainly of chronic viral infection [33], [34]. Carpentier et al. showed that following liver transplantation, patients with high T-regs frequencies were more likely to have aggressive HCV recurrence [12]. In a mouse model of herpes simplex virus associated retinitis, T-regs were associated with progressive and more severe tissue invasive disease [35]. The role of T-regs in controlling CMV-specific immune responses was recently examined. In blood samples obtained from healthy donors and transplant recipients, depletion of T-reg cells from PBMCs resulted in an enhanced CMV-specific CD4^+^ and CD8^+^ T-cell response *in vitro* [36], [37].

The accumulation of virus-specific T-regs during virus replication could be due to the expansion of pre-existing populations of thymus-derived ‘natural’ T-regs that are specific for viral antigens; or it could also reflect the *de novo* generation of ‘induced’ T-regs from naive virus-specific CD4+ T cells. An important factor in the generation of ‘induced’ T-regs appears to be TGF-β [38], [39]. There are limited *in vitro* data for the role of CMV-proteins in the regulation of T-reg generation. In a co-culture model of T-cells and murine-CMV infected fibroblasts, an up-regulation of TGF-β and IL-10 expression was observed which subsequently induced T-cell differentiation to T-regs [40]. Our study is unable to answer in detail the question as to whether CMV-replication influences T-reg development or function. Nevertheless, the observed correlation between T-reg frequency at baseline and viral-load quantification over the ensuing two weeks (R^2^ = 0.61) would suggest that T-regs may play an important role in regulating CMV-replication. The role of Th-17 responses during viral infection are poorly characterized, but virus-specific IL-17-producing CD4+ T-cells have been detected in mice following infection with murine-CMV, herpes simplex virus, and influenza virus [15], [16], [17]. The generation of polarized Th-17 cells during viral infection has been correlated with high levels of IL-6 and may also be influenced by transforming growth factor-β (TGF-β) [41]. Th-17 cells are implicated in driving harmful inflammation during autoimmunity, and IL-17 may contribute to immunopathology during host responses against viruses. In our study, we did not find Th-17 cell frequencies correlated with CMV outcomes.

Our study has several limitations. First the sample size is small and includes a heterogeneous population of solid organ transplant recipients. We tried to minimize this effect by studying two very distinct groups of patients (those who required anti-viral treatment compared with those who spontaneously cleared viremia). However, until these results are confirmed in a larger subset of patients, this study should be considered preliminary, since the effect of co-factors such as transplant type, immunosuppressive regimen and D/R serostatus may only be apparent with larger sample sizes. These factors were not significant predictors of outcome in this group although the immunosuppression used was relatively homogenous. In a study of 259 patients with CMV disease, neither D/R status nor transplant type had an effect on viremia clearance [42]. The intensity of immunosuppression had a modest effect on time to viral clearance but showed no association with overall clearance rates or recurrence [43]. Secondly it would be interesting to measure CMV-specific T-regulatory and CMV-specific Th-17 cells. Unfortunately, the frequency of these cells is very low in peripheral blood and *in vitro* cultivation could well generate an expansion bias. Thirdly, the precise effects of T-regs on CMV-specific T-cells can be assessed in various ways. We chose to evaluate the frequencies of CMV-specific CD4^+^ and CD8^+^ T-cells by using interferon gamma as the predominant cytokine produced in response to specific stimulation. Production above background was used to identify responsive virus-specific T-cells (>0.2%). However, the functional impact of T-regs could primarily affect other markers such as interleukin 2 or T-cell proliferation.

Despite these limitations, our study provides novel insight into T-reg and Th-17 cell dynamics in transplant recipients with active CMV-replication. CMV-specific T-cell dynamics were analyzed in conjunction with virologic parameters and clinical outcomes. T-regs frequencies are elevated in patients developing CMV-disease when compared with those who spontaneously clear CMV-viremia. High CMV-specific CD4^+^ T-cells and low T-regs counts were significantly associated with clearance of viremia and protection from relapse. Larger multicenter studies are required to further explore the predictive value of CMV-specific T-cell and T-reg monitoring after transplantation. This may help individualize patient management. Immune monitoring could differentiate between patients at risk for CMV-replication and those protected, thereby informing critical treatment decisions, reducing morbidity and ultimately preserving graft function.

## Supporting Information

Figure S1 **Study design.** Patients were enrolled for immune monitoring within 3 days after detection of CMV-replication. At this initial time-point CMV-specific CD4^+^ and CD8^+^ T-cells were measured, as well as absolute frequencies of CD4^+^CD25^+^FoxP3^+^ T-regulatory cells and IL-17 producing CD4^+^ T-cells. In patients who commenced anti-viral treatment, we measured the same immune markers at the end of anti-viral treatment and 1 month later.
(TIF)

Figure S2 **Dynamic aspects of CMV-specific CD4^+^ and CD8^+^ T-cells, and T-regs in patients with increasing versus decreasing CMV-specific CD4^+^ T-cells.** Square (□) indicates CMV-specific CD4^+^ T-cell response. Circle (○) indicates CMV-specific CD8^+^ T-cell response. Triangle (▵) indicates T-reg response. Black bar indicates median value, whiskers indicate interquartile range. **A**, patients with increasing CMV-specific CD4^+^ T-cell responses. **B**, patients with decreasing CMV-specific CD4^+^ T-cell responses.
(TIFF)
