# Supplementary figures and images for: An Analysis of Regulatory T-Cell and Th-17 Cell Dynamics during Cytomegalovirus Replication in Solid Organ Transplant Recipients
Source: PLoS One. 2012 Oct 11;7(11):e43937. doi: 10.1371/journal.pone.0043937 (PMC3469568; doi:10.1371/journal.pone.0043937)

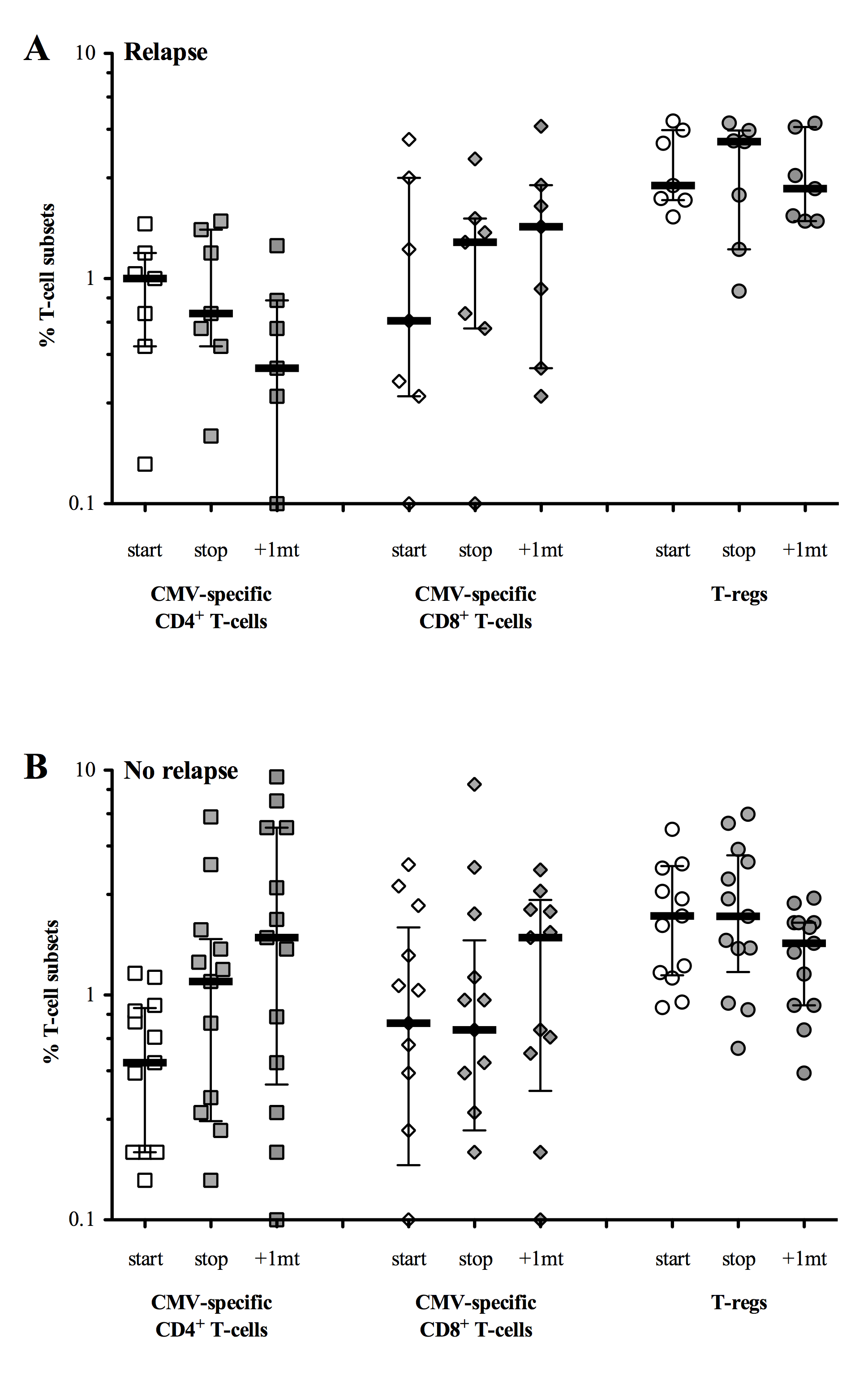

Supplement: Figure S2 — Dynamic aspects of CMV-specific CD4+ and CD8+ T-cells, and T-regs in patients with increasing versus decreasing CMV-specific CD4+ T-cells. Square (□) indicates CMV-specific CD4+ T-cell response. Circle (○) indicates CMV-specific CD8+ T-cell response. Triangle (▵) indicates T-reg response. Black bar indicates median value, whiskers indicate interquartile range. A, patients with increasing CMV-specific CD4+ T-cell responses. B, patients with decreasing CMV-specific CD4+ T-cell responses. (TIFF) [file pone.0043937.s002.tif]
